# Apparent Bicarbonate Space in Children

**DOI:** 10.1100/tsw.2006.32

**Published:** 2006-01-26

**Authors:** Horacio A. Repetto, Roberto Penna

**Affiliations:** ^1^ Service of Pediatrics, Hospital Nacional Prof. A. Posadas, Buenos Aires, Argentina; ^2^ Intensive Care Unit, Hospital Nacional Prof. A. Posadas, Buenos Aires, Argentina; ^3^ Department of Pediatrics, Faculty of Medicine, University of Buenos Aires, Argentina

**Keywords:** bicarbonate (HCO_3_–), bicarbonate space, metabolic acidosis, correction, buffer mechanisms, acidosis treatment, children acid-base derangements

## Abstract

The amount needed to change the concentration of a solute requires the knowledge of its volume of distribution in the solution. Electrolytes that do not participate in active metabolic reactions have a fixed volume of distribution that corresponds to the volume of water in which they solubilize. Bicarbonate infusion is used to correct hyperchloremic metabolic acidosis. Its volume of distribution (bicarbonate space) changes with its participation in the blood buffer systems. In other words, it is not a fixed physical volume, like that of other solutes. In this paper, we shall review experimental studies that supported evidence for this knowledge and analyze the basic hypothesis to explain the phenomena. Since we have not found clinical studies in children, we shall report our experience in a group of patients with metabolic acidosis treated with bicarbonate infusion in whom apparent bicarbonate space was measured and compared with data in adults from the literature. Guidelines for amount of bicarbonate needed to increase its concentration according to baseline bicarbonate concentration will be suggested.

The intravenous administration of sodium bicarbonate (Na $HCO_3$) is used in some cases of metabolic acidosis to increase its concentration and improve the change in free hydrogen ion concentration. The amount given to produce a determined increase in concentration is calculated with the formula that relates amount of solute to concentration

$$([\ ])\text{ and volume of solvent : amount} = \text{concentration} \times \text{volume.}$$

When the salt given does not participate in any immediate metabolic reaction, the volume of distribution is that of the water in which the salt is solubilized. In the case of NaCl, the apparent volume is increased because water is translocated from the intracellular to the extracellular space alongside with the increase in extracellular tonicity induced by the administered salt.

The first studies in experimental animals showed that the distribution volume for $HCO_3^-$ salts was approximately twice the extracellular fluid (ECF) volume (40%)[1]. The same distribution was observed in normal individuals[2].

Schematically, bicarbonate can be considered to distribute between two different spaces: (1) the dilutional space (DS), where it is freely dissolved: the extracellular water, the fraction entering the erythrocytes, and a small amount dissolved in some soft tissues and bone; (2) the titration space (TS), accounting for that amount of bicarbonate titrated by nonbicarbonate buffers (NBB): plasma proteins (albumin circulates like a polyanion), dibasic phospate, hemoglobin. (Due to it low pK, the anions of sulfuric acid and the organic acids encompassing the plasma anion gap cannot buffer acids entering the ECF.) Garella et al. observed that some patients with severe metabolic acidosis and very low plasma bicarbonate concentrations needed amounts of $HCO_3^-$ larger than expected[3]. They evaluated the effects of the infusion of sodium bicarbonate in dogs with low, normal, and high bicarbonate concentration, and they found significant differences in the increase in concentration between the acidotic and both the normal and alkalotic animals. The calculated mean "apparent" bicarbonate space was equivalent to 88% of body weight in acidotic dogs, as opposed to 50 and 44%, respectively, in normal and alkalotic dogs. The authors speculate that this effect depends on the efficiency of a buffer pair, determined both by its pK (better when it is closer to the pH of the solution) and by its concentration. When bicarbonate concentration decreases during the development of metabolic acidosis, other buffers would progressively increase their relative participation in the buffering of the acid load. Since the extracellular compartment is relatively poor in NBB, this increased participation must take place also in the intracellular space. In the reverse situation, when bicarbonate is infused, its increase in concentration would augment its buffering activity in the extracellular compartment, titrating acids previously titrated by NBB.

In 1983, Adrogué et al.[4] provided evidence that the enlargement of the bicarbonate space depends on the low level of bicarbonate itself and is not a consequence of acidemia. In a carefully designed study, they acutely infused sodium bicarbonate to five groups of intact dogs, previously generating:

- Chronic metabolic acidosis (mean $[HCO_3^-]$ = 13.5 m*M*/l; mean pH = 7.20),
- Chronic respiratory alkalosis (mean $[HCO_3^-]$ = 14.8 m*M*/l; mean pH = 7.40),
- Chronic respiratory acidosis (mean $[HCO_3^-]$ = 35.8 m*M*/l; mean pH = 7.28), and
- Chronic metabolic alkalosis (mean $HCO_3^-]$ = 37.4 m*M*/l; mean pH = 7.47)

trying to achieve a broad range of steady-state plasma bicarbonate concentrations in association with acidemia, alkalemia, and normal pH. By measuring concomitantly the ECF volume and correcting $[HCO_3^-]$ and pH for the increased $PaCO_2$ (which titrates NBB) generated by the acute infusion, they were able to calculate the bicarbonate freely dissolved in the ECF volume, that one entering the ICF (including erythocytes) and that one titrated by hemoglobin and plasma proteins. Plasma unmeasured anion concentration (anion gap) was not significantly altered by bicarbonate infusion in any of the experimental groups. Pooled data from all the studies showed an inverse correlation between initial plasma $[HCO_3^-]$ and the apparent space of distribution of the infused bicarbonate, independent of whether the decreased $[HCO_3^-]$ was of respiratory or metabolic origin. Consequently, correlation with initial plasma pH was positive in the respiratory and negative in the metabolic studies. There was no difference in the measured extracellular volume between the five groups to account for the different "bicarbonate space". Thus, the influence of initial $[HCO_3^-]$ must have been a manifestation of variable bicarbonate removal from that compartment. The authors hypothesize that the observed differences resulted from the different range of pH over which NBB were titrated in the various states of acid-base equilibrium studied, assuming that changes in intracellular pH roughly parallel changes achieved by the infusion in the extracellular space.

Fernández et al.[5] analyzed the data reported by Adrogué et al.[4] establishing the dependence of the ABS (apparent bicarbonate space) on the initial plasma $[HCO_3^-]$. They used chemical principles of buffer systems to describe a model integrating the chemical and physiological behavior of biological buffers: nonvolatile buffers and the $HCO_3^-/CO_2$ system, open to the atmosphere.

In a closed system, the buffering power (capacity to protect the pH when $H^{\%}$ or $OH^-$ is added) is maximal when the buffer is at its pK (pH at which 50% is dissociated and 50% undissociated). In an open system like $HCO_3^-/CO_2$, the amount of total $CO_2$ changes because $[HCO_3^-]$ changes when $H^+$ or $OH^-$ is added, but dissolved $CO_2$ remains stable due to ventilation. The buffering power of this system increases linearly with $[HCO_3^-]$ and is independent of pH, since pH can be varied over a wide range by modifying $pCO_2$.

When both systems coexist, added $H^+$ or $OH^-$ are titrated by both types of buffers. The amount titrated by either system is proportional to the absolute buffering power of each system. So, depending on the bicarbonate concentration, its buffering power can be greater than the nonbicarbonate one, titrating most of the added acid or base; but, on the other hand, when its concentration decreases, a larger proportion of acids or bases will be titrated by the NBB. Based on these concepts, the Apparent Bicarbonate Space should be the sum of the Dilution Space (DS) plus the Titration Space (TS); and the TS should be directly proportional to the buffering power of NBB and inversely proportional to the buffering power of bicarbonate (dependent on its concentration). Utilizing data in normal humans from Garella et al. and estimating the buffer power for the two systems, the authors develop a formula to calculate the ABS based on initial $[HCO_3^-]$:

$$ABS = \{0.4\ (DS) + 2.6/[HCO_3^-]\} \times \text{body weight in kg}$$

It is interesting to notice how similar this formula is to the empiric equation obtained by Adrogué et al. from their experiments in dogs (pooled data of Fig. 2 in Adrogué et al.[4]):

$$ABS = \{0.36 + 2.44/[HCO_3^-]\} \times \text{body weight}$$

Before Garella group's report, we had noticed that the use of the "classical" formulae stated in many books and hospital guidelines (bicarbonate amount to be administered = difference in concentration × 30 or 40% of body weight) led to lesser increases in $[HCO_3^-]$ than predicted. We then decided to study the bicarbonate space in children with metabolic acidosis requiring bicarbonate infusions according with our regular protocols. We followed Adrogué's demonstration that the bicarbonate space was dependent on the initial bicarbonate concentration and infused different amounts with a predetermined table relating the ABS to the initial $[HCO_3^-]$ and calculated the volume with the equation that relates the three variables of a solution: vol = amount/concentration.

Patients were eligible when they (1) had clinical dehydration (DHT); (2) had metabolic acidosis that, according to the staff, would benefit with the infusion of bicarbonate; (3) had no concomitant respiratory disease; (4) did not require assisted mechanical ventilation; and (5) $pCO_2$ levels were adequate to predicted respiratory compensation (Table 1).

Physicians and nurses taking care of the patients were informed of the protocol and participated in the clinical follow-up. Informed consent was required of the parents.

The calculated increase in $[HCO_3^-]$ was discussed with the attending physician and it was between 5 and 10 mEq/l (mean 8.13 mEq/l). The volume of distribution chosen for the calculation, following Adrogue et al.[4]., was between 40 to 90% of BW depending on the initial level (median 61%)(Table 2).

We infused 0.5 *M* bicarbonate over 60 min, dissolving 1 *M* solution in distilled water or 5% DW depending on the initial glycemia; 30 min after the end of the infusion, laboratory was obtained and the volume of distribution was calculated. Measured volume (MVD) = amount infused ÷ difference in concentration. The theoretical ABS was calculated introducing the initial $[CO_3H^-]$ in the formulae developed by Fernandez et al.[5].

We found a significant inverse correlation between measured bicarbonate volume of distribution and initial $[CO_3H^-]$ r= -0.62; $p < 0.025$ (Fig. 1). Initial pH showed an inverse weakly correlation with $[CO_3H^-]$ r = 0.47; $p = 0.05$. There was a very good correlation comparing our measured bicarbonate volume of distribution with Fernandez et al.[5]. ABS $(0.4 + 2.6/ HCO_3^-]$ r = 0.67; $p < 0.01$ (Fig. 2).

In conclusion, we confirmed the findings of Garella et al.[3] regarding the different volumes of distribution of infused bicarbonate in relation to the initial $[CO_3H^-]$ and the certification by Adrogué et al.[4] of its dependence on the initial $[CO_3H^-]$ and not on pH. We were able to draw a correlation curve to obtain the approximate value of distribution of infused bicarbonate in percentage of BW from the initial $[CO_3H^-]$ (Fig. 1).

**TABLE 1**
**Individual Patient Data***

| Pt. Nr. | Age (Mo.) | M/F | Weight (kg) | DHT (%BW ) | pH In (mEq/l) | pH Fi (mEq/l) | Bic In (mEq/l) | Bic Fi (mEq/l) | Δ Bic Prog. (mEq/l) | Δ Bic Obtain. (mEq/l) | Bic.Infus (mEq/kg) | Vol. Dist. Est. (%) | Vol. Dist. Meas. (%) | Vol Dist. Calc. (%) |
|---|---|---|---|---|---|---|---|---|---|---|---|---|---|---|
| 1 | 5 | M | 4.3 | 7 | 7.14 | 7.37 | 7.4 | 19.1 | 10 | 11.7 | 6.9 | 70 | 60 | 75 |
| 2 | 7 | F | 6.5 | 6 | 7.39 | 7.44 | 13.6 | 19.5 | 5 | 5.9 | 3 | 60 | 51 | 59 |
| 3 | 4 | M | 6.5 | 7 | 7.27 | 7.32 | 75.3 | 18.9 | 5 | 3.3 | 2 | 40 | 60 | 57 |
| 4 | 3 | M | 4.5 | 5 | 7.25 | 7.39 | 11.5 | 19.4 | 7 | 7.9 | 3.5 | 50 | 44 | 63 |
| 5 | 6 | F | 6 | 6 | 7.20 | 7.39 | 10.4 | 20 | 10 | 9.6 | 5 | 50 | 52 | 65 |
| 6 | 7 | M | 5.9 | 6 | 7.18 | 7.44 | 9.1 | 18.5 | 10 | 9.4 | 6 | 60 | 64 | 68 |
| 7 | 6 | M | 5.8 | 10 | 7.20 | 7.44 | 5.3 | 12.3 | 10 | 7 | 6 | 60 | 83 | 89 |
| 8 | 1 | M | 3.5 | 10 | 7.29 | 7.46 | 9.3 | 16.7 | 10 | 7.4 | 6 | 60 | 81 | 69 |
| 9 | 5 | M | 5.5 | 8 | 7.25 | 7.38 | 9.5 | 19 | 10 | 9.5 | 7 | 70 | 73 | 68 |
| 10 | 7 | F | 8 | 8 | 7.26 | 7.37 | 13.3 | 18.5 | 7 | 5.2 | 4.2 | 60 | 81 | 59 |
| 11 | 11 | F | 8.5 | 9 | 7.23 | 7.40 | 11.8 | 20.5 | 10 | 8.7 | 5 | 50 | 57 | 62 |
| 12 | 6 | F | 5.7 | 8 | 7.22 | 7.39 | 9.7 | 19 | 10 | 9.3 | 6 | 60 | 64 | 67 |
| 13 | 9 | F | 7 | 9 | 7.27 | 7.41 | 14.3 | 19 | 5 | 4.7 | 2.5 | 50 | 53 | 58 |
| 14 | 10 | F | 8.8 | 5 | 7.23 | 7.40 | 11.2 | 19.3 | 10 | 9.1 | 5 | 50 | 55 | 63 |
| 15 | 2 | F | 4.3 | 12 | 7.10 | 7.38 | 7.7 | 17 | 10 | 9.3 | 7 | 70 | 75 | 74 |
| 16 | 5 | M | 5.4 | 9 | 7.25 | 7.34 | 12.5 | 17 | 7 | 4.5 | 2.8 | 40 | 62 | 61 |
| 17 | 7 | M | 6.8 | 10 | 7.16 | 7.34 | 4.6 | 11.6 | 10 | 7 | 8 | 80 | 114 | 96 |
| 18 | 11 | M | 9.1 | 7 | 7.17 | 7.36 | 9.1 | 17 | 7 | 7.9 | 5.6 | 80 | 70 | 69 |
| 19 | 4 | M | 5.7 | 5 | 7.30 | 7.40 | 9.5 | 14.9 | 5 | 5.4 | 2.5 | 50 | 46 | 97 |
| 20 | 48 | M | 16 | 5 | 7.22 | 7.40 | 8.5 | 14.5 | 5 | 6 | 3.5 | 70 | 58 | 71 |
| 21 | 4 | F | 4.2 | 11 | 7.12 | 7.50 | 4.4 | 17.4 | 10 | 13 | 9 | 90 | 70 | 99 |
| 22 | 12 | F | 10 | 8 | 7.18 | 7.35 | 10 | 18 | 7 | 8 | 4.2 | 60 | 52 | 66 |
| 23 | 5 | F | 5.8 | 7 | 7.22 | 7.40 | 13 | 17.5 | 5 | 4.5 | 2.5 | 50 | 55 | 60 |
| 24 | 9 | F | 8 | 11 | 7.15 | 7.30 | 8 | 17.8 | 10 | 9.8 | 7 | 70 | 71 | 72 |
| 25 | 18 | F | 10 | 7 | 7.16 | 7.37 | 7 | 16 | 10 | 9 | 7 | 70 | 77 | 77 |
| 26 | 12 | F | 9.5 | 6 | 7.25 | 7.42 | 12 | 18 | 7 | 6 | 3.5 | 50 | 58 | 62 |
| 27 | 48 | F | 15 | 8 | 7.28 | 7.42 | 14.3 | 19.2 | 5 | 4.9 | 3 | 60 | 61 | 58 |
| 28 | 3 | M | 4 | 9 | 7.15 | 7.30 | 6.6 | 16.8 | 10 | 10.2 | 8 | 80 | 78 | 79 |
| 29 | 36 | F | 12 | 6 | 7.28 | 7.35 | 14.2 | 21.5 | 7 | 7.3 | 3.5 | 50 | 48 | 58 |
| 30 | 13 | M | 10 | 7 | 7.12 | 7.31 | 2.7 | 12.1 | 10 | 9.4 | 8 | 80 | 85 | 114 |

*30 children (median age 11 months, range 1–48 months; 60% males; average weight 7.65 kg; average estimated degree of DHT 7.73% of BW); initial pH 7.21 (median, range 7.10–7.39), initial [$CO_3H^-$] varied between 2.7–15.3 mEq/l (median 7.21).

DHT: estimated dehydration; pH In: initial pH; pH Fi: pH after infusion; Bic In and Bic Fi: idem pH; Δ Bic. Prog.: programmed increased in bicarbonate concentration; Δ Bic. Obtain.: increase obtained; Vol. Dist.: volume of distribution; Est.: estimated; Meas.: measured; Calc.: calculated with Equation [ref. 5], all as % BW.

Moreover, when comparing our measured bicarbonate space with the one theoretically calculated by Fernandez et al.[5] for adults, we found that there was an excellent correlation. Consequently, we believe that their formulae can be utilized in calculating the amount of bicarbonate infused to obtain a predicted change in concentration.

**TABLE 2**
**Estimated Volume of Distribution (ABS)**

| Initial Bicarbonate (mEq/l) | Estimated Volume (%BW) |
|---|---|
| >15 | 40% |
| 12.5–15 | 50% |
| 10–12.4 | 60% |
| 8–9.9 | 70% |
| 6–7.9 | 80% |
| 4–5.9 | 90% |
| <4 | 100% |

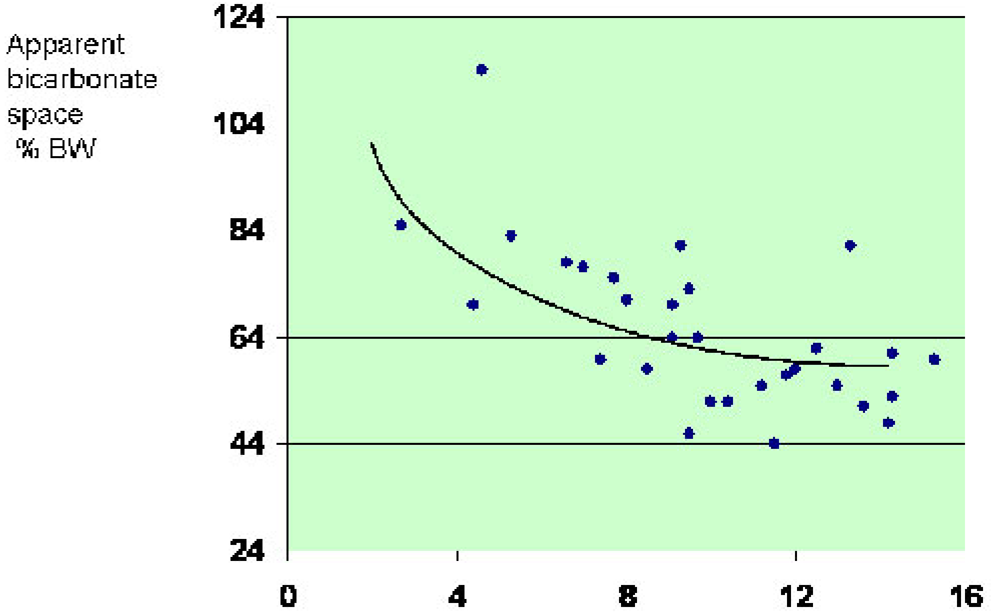

**FIGURE 1.** Fig 1. Initial bicarbonate concentration in mEq/L

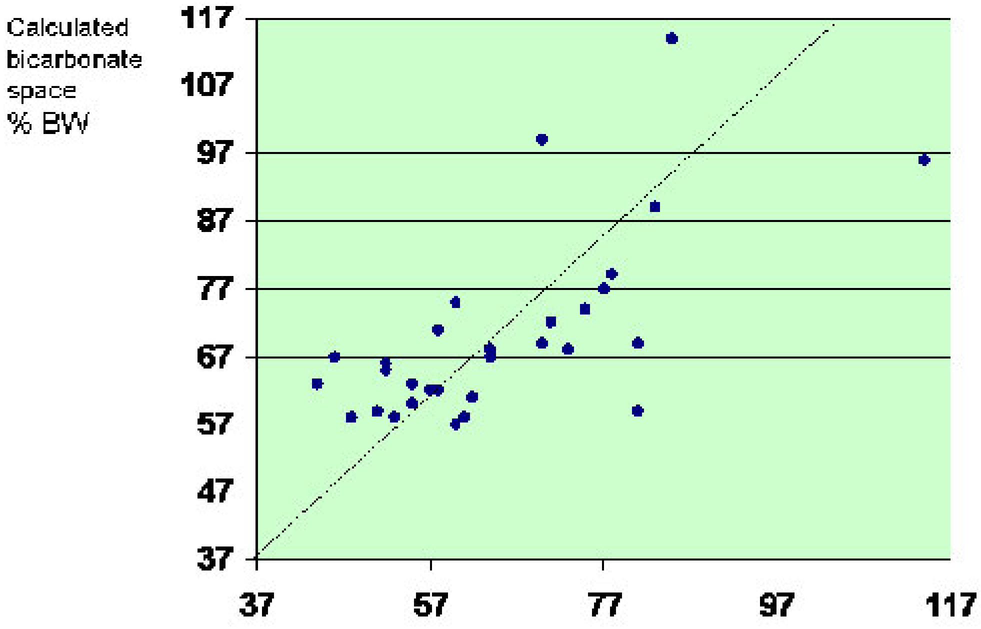

**FIGURE 2.** Measured bicarbonate space. % BW.

## ACKNOWLEDGMENTS

To the physicians and nurses who took care of the patients.
